# Opening Address

**Published:** 1907-01

**Authors:** 


### OPENING ADDRESS.

By the President of the South Carolina State Dental Society. 1906.

It is with extreme gratification and pleasure that I observe so many in attendance upon this, our thirty-sixth annual meeting. It is a pleasure to greet you upon this occasion, and it is with a whole-hearted wish and desire that we may each derive profit, and an abundance of enjoyment, during this week.

No more fitting introduction to an address of this kind can be imagined than that used by Dr. Frank Holland, president of the Georgia State Dental Society, who said in part, and which sentiment I echo: "It shall be my honest endeavor to be fair and just to all, at the same time you must realize that unless the rules are enforced, the measure of success and dignity which we all desire cannot be attained. I beg that you be patient and tolerant, and that your criticisms be tempered with mercy, allowing me credit for honesty of purpose and a strong desire for the welfare of our organization."

Manifestly the reason of the custom for the president to deliver an address at the end of his term of office is to permit the presiding officer to render an occount of his services to the Association, and to make such suggestions and recommendations as in his opinion may enhance its interests, promote its prosperity and extend its influence.

With this conception of my duty in view, I will not on the present occasion endeavor to weave garlands of florid expressions with which to adorn our transactions, but rather will attempt to bring to your attention such matters as during the past year have suggested themselves to me as likely to promote the betterment of our organization, and with your indulgence I will present these suggestions.

The life and usefulness of our Association is dependent upon its membership. This is axiomatic in its clearness, and needs no explanation. But as the most common and apparent things are overlooked or neglected, I desire to say one word about membership.

It is true that during the past years of our existence as a dental society we have accomplished many things which have tended to put our organization on a high plain, and which have produced marked results, both in extending the influence of our body and in increasing our internal strength; but for fear that we may become careless and over confident, let me urge upon each one present the need to be constantly vigilant and ever ready to see and to do everything possible to swell the ranks of our society, that not one man in South Carolina who is qualified to practice dentistry within the borders of our state can be found whose name does not appear on our roll of members.

Individual effort can accomplish much, therefore as individuals exert yourselves, but we should have some systematic plan for the promotion of this feature of our work. I recommend that we have a committee, selected as seems best to the Association, whose duty it shall be to inquire into the matter of membership and to take such steps as are necessary for the enlargement of our society. By co-operating with the individual members of this body, this committee could greatly extend our ranks, and would be efficient in bringing the indifferent into the Association.

Directly associated with the matter of membership, is that of the disregard in this state, by some dentists, of the laws governing the practice of dentistry, and I take it, that it is incumbent upon the South Carolina State Dental Association to consider this evil and to adopt some course which will tend to remedy this unfortunate state of affairs.

The people of this state do not understand the gravity and importance of unethical practice, and it is largely due to this fact that some dentists are now openly defying our board and the authority of the law.

It is not that the law is inadequate in its provisions to protect society and the dental profession from the encroachments of unscrupulous men, but rather this evil exists because, as stated above, the matter is not fully understood.

Some of these dentists who have been practicing unlicensed and hence unlawfully, have been reported by the board to the grand juries of our several counties where the offenses have been committed, but in each instance the bill has been thrown out and I ascribe the reason for this non-action to the fact that grand jurors, who are selected from the qualified voters, do not realize the importance of the matter, and for the additional reason that there has never been any one to properly present the case and push the matter.

To remedy this, I would suggest that this Association, through its board, employ counsel to take charge of these cases, and whenever a dentist is found practicing unlicensed and contrary to law, to prosecute him vigorously.

If need be a detective should be employed to work up evidence, so that a grand jury could have some facts on which to return a true bill. Perhaps detectives would not always be necessary, but in case the circumstances warranted such a course of action I suggest that they be employed.

This Association through its board should do this much needed work. Should see that the law is enforced whenever any violation of it is brought to their attention, for the duty should not be allowed to rest upon local dentists of this Association to personally prosecute offenders. Local dentists should report cases of unlawful practice to the board; the board should employ counsel to prosecute to the limit of the law any person who flagrantly violates the provisions of the dental statutes of this state.

The cost of employing counsel would not be over fifty dollars for each case, and I firmly believe that only one prosecution would be necessary, because all others who subsequently might be tempted to practice dentistry with no license would be deterred therefrom by the moral effect which would be produced by the former prosecution.

I further recommend that should an alleged offender be found guilty, that he be licensed only after passing satisfactorily the regular examination of the State Board of Dental Examiners.

With your indulgence. I desire to say that I heartily recommend the practice of giving clinics. Many of us profit by the experience of others, and it is a duty our more fortunate brothers owe to the profession at large to demonstrate any special line of work with which he may have had success. The same might be said of the publication of articles in regard to cases of special interest.

Perhaps the plan of publishing papers does not appeal to some, and I therefore suggest that they read their papers before this Association, instead of publishing them. This is at present the custom, and in saying this I do so with a view to encourage the practice, for I believe we will reap rich rewards from it.

The occasion of our annual meeting is a time when we may enjoy friendly intermingling with each other. I hope that each one present will take advantage of this opportunity and by so doing add to the enjoyment of others, and reap a pleasant harvest for one's self.

It is customary for a retiring officer to express his gratitude at the honor conferred upon him by the elevation of himself to the position of president, and it is with all sincerity and in truth that I adhere to this custom, for it has been a pleasure to occupy the position of president of the South Carolina State Dental Association, and it is an honor which I thoroughly appreciate and for which I thank you.

DISCUSSION.

DR. CRENSHAW: In his address the president touches on two points that can be made of very great advantage to the Association. You have a liberal policy, and one which if concurred in by all the members of your Association you ought to be able to bring into the Association every young man who comes before this board. I don't know of anything more helpful to the young men themselves than to get into their state association. A young man just starting out with his license is very likely to get into trouble, to go into the Dental Parlor, which is the greatest menace to the profession of dentistry. I once advertised, thirty years ago, and have never forgiven myself for it, or forgotten it. If any measure could be found to keep young men out of the Dental Parlors, they will accomplish the greatest good to the young men that can possibly come to them. The other point touched on is that of stopping illegal practice—violations against the law regulating the practice of dentistry in your state. In our own state only last week we organized a Protective Association, and I believe great good is going to come out of it. The members of our Association, as many as will join, will pay an initiation fee which goes into the treasury, and if a violation of the law comes within your influence the case is followed up by this Association, and in the strength that comes in the combination it may be that these violations may be speedily stopped, but if it comes within the influence of a practitioner, a member of this combination and not of the Association, then he pays $5.00 to become a member because he has waited until his own territory is invaded before he puts his influence with that of the protective association. I believe the two points touched upon are very properly raised in the address.

Dr. Smith: Dr. Crenshaw has brought up some interesting matter and I think it is just as well that we appoint a committee to look over those particular points in the paper and report to the Association.

